# Commentary: *Toxoplasma* depends on lysosomal consumption of autophagosomes for persistent infection

**DOI:** 10.3389/fmicb.2017.01876

**Published:** 2017-09-26

**Authors:** Giovanni Di Guardo

**Affiliations:** Faculty of Veterinary Medicine, University of Teramo, Teramo, Italy

**Keywords:** *Toxoplasma gondii*, autophagy, lysosomes, infection, neuropathogenesis

*Toxoplasma gondii*, a protozoan agent infecting several mammalian species, is responsible for almost 2 billion cases of human infections worldwide, with severe *T. gondii*-related/associated disease also occurring in immunocompromised hosts (Montoya and Liesenfeld, 2004).

Noteworthy, a key role for proteolysis has been recently demonstrated within *T. gondii* lysosomal organelle (Di Cristina et al., 2017). Indeed, the aforementioned paper reports that disruption of a cysteine protease enzyme located in the protozoan's vacuolar compartment (VAC) results in a strong reduction of *T. gondii* chronic neural infection, with previous accumulation of undigested autophagosomes in the parasitic cytoplasm (Di Cristina et al., 2017).

Albeit of significant relevance and also of potential therapeutic value toward this major zoonotic pathogen, the possibility that inhibiting *T. gondii* VAC-associated autophagic machinery may adversely affect the host's innate immunity against the infection should be seriously taken into account. In this respect, it has been demonstrated that all three genotypes of *T. gondii* elicit the production of “neutrophil extracellular traps” (NETs) on behalf of murine and human polymorphonuclear (PMN) leukocytes, both *in vitro* and *in vivo* (Abi Abdallah et al., 2012). These neutrophil-released components, the formation of which may be equally induced by other protozoan agents, such as *Leishmania amazonensis* (Guimarães-Costa et al., 2009) and *Plasmodium falciparum*, (Baker et al., 2008), have been shown to exert a powerful anti-microbial defense activity also against bacterial (Brinkmann et al., 2004) and fungal pathogens (Urban et al., 2009). Furthermore, NET release by PMN cells does not appear to depend upon active *T. gondii* invasion, nor upon host cell phagocytic function (Abi Abdallah et al., 2012), which could be also negatively impacted, in turn, by the aforementioned inhibition of *T. gondii* VAC-associated autophagic pathway (Di Cristina et al., 2017). Indeed, beside DNA and histones NETs contain several antimicrobial factors mimicking those normally found in PMN lysosomal compartment, like bacterial permeability-increasing protein (BPI), myeloperoxidase and neutrophil elastase (Brinkmann et al., 2004).

On the basis of the above, we cannot exclude *a priori* that the inhibition of *T. gondii*-dependent lysosomal machinery may result in a concurrent suppression of the aforementioned innate immune response mechanisms ensuring an efficient anti-protozoan defense.

Additional caution should be taken when dealing with the comparative neuropathology and neuropathogenesis of *T. gondii* infection, which could vary across different mammalian hosts. In this respect, while the cerebral inflammation's degree and severity in *T. gondii* meningoencephalitis-affected striped dolphins (*Stenella coeruleoalba*) (Di Guardo et al., 2010) has been shown to be positively correlated, on one side, with the brain tissue levels of 5-lipoxygenase (5-LOX) (Di Guardo et al., 2015)—a key enzyme of the eicosanoid pathway mediating inflammatory reactions (Pihlaja et al., 2017)—an excessive proinflammatory response has been reported, on the other side, in the brains of 5-LOX-deficient mice, which also succumbed to *T. gondii* infection at the early onset of chronic disease, following the development of severe encephalitic lesions (Aliberti et al., 2002).

In conclusion, with the aim of clarifying the open issues outlined above, further studies in both spontaneous and experimental disease models are needed, with special emphasis on *T. gondii* infection's neuropathogenesis and mutual host-parasite relationships.

## Author contributions

After having carefully read the recent *Nature Microbiology* article by Dr. Manlio Di Cristina and coworkers, upon which this manuscript is based, the Author (GDG) has autonomously and independently written the present Commentary.

### Conflict of interest statement

The author declares that the research was conducted in the absence of any commercial or financial relationships that could be construed as a potential conflict of interest.
